# Supplementary material for: Virus adaptation to heparan sulfate comes with capsid stability tradeoff
Source: eLife. 2024 Dec 23;13:e98441. doi: 10.7554/eLife.98441 (PMC11717363; doi:10.7554/eLife.98441)
Supplement: Supplementary file 1. — ΔΔG<0: Destabilizing mutation. ΔΔG>0: Stabilizing mutation. [file elife-98441-supp1.docx]

**Supplementary figure 1**

| **Mutation** | **pH** | **Temperature (°C)** | **Predicted free Gibbs energy change value (ΔΔG)** |
| --- | --- | --- | --- |
| VP1-L97R | 7 | 25 | -0.65  (Destabilizing) |
|  | 5 | 25 | -0.70  (Destabilizing) |
|  | 7 | 55 | -0.51  (Destabilizing) |
| VP1-E167G | 7 | 25 | -1.15  (Destabilizing) |
|  | 5 | 25 | -1.10  (Destabilizing) |
|  | 7 | 55 | -0.74  (Destabilizing) |
| VP1-E145Q | 7 | 25 | -0.80  (Destabilizing) |
|  | 5 | 25 | -0.89  (Destabilizing) |
|  | 7 | 55 | -0.71  (Destabilizing) |
